# Metabolic flux analysis to increase oil in seeds

**DOI:** 10.1093/plphys/kiae595

**Published:** 2024-11-05

**Authors:** Thiya Mukherjee, Shrikaar Kambhampati, Stewart A Morley, Timothy P Durrett, Doug K Allen

**Affiliations:** Donald Danforth Plant Science Center, 975 North Warson Road, St. Louis, MO 63132, USA; Salk Institute for Biological Studies, 10010 N Torrey Pines Rd, La Jolla, CA 92037, USA; Donald Danforth Plant Science Center, 975 North Warson Road, St. Louis, MO 63132, USA; Department of Biochemistry and Molecular Biophysics, Kansas State University, 1711 Claflin Rd, Manhattan, KS 66502, USA; Donald Danforth Plant Science Center, 975 North Warson Road, St. Louis, MO 63132, USA; United States Department of Agriculture, Agriculture Research Service, 975 North Warson Road, St. Louis, MO 63132, USA

## Abstract

Ensuring an adequate food supply and enough energy to sustainably support future global populations will require enhanced productivity from plants. Oilseeds can help address these needs; but the fatty acid composition of seed oils is not always optimal, and higher yields are required to meet growing demands. Quantitative approaches including metabolic flux analysis can provide insights on unexpected metabolism (i.e. when metabolism is different than in a textbook) and can be used to guide engineering efforts; however, as metabolism is context specific, it changes with tissue type, local environment, and development. This review describes recent insights from metabolic flux analysis in oilseeds and indicates engineering opportunities based on emerging topics and developing technologies that will aid quantitative understanding of metabolism and enable efforts to produce more oil. We also suggest that investigating the key regulators of fatty acid biosynthesis, such as transcription factors, and exploring metabolic signals like phytohormones in greater depth through flux analysis could open new pathways for advancing genetic engineering and breeding strategies to enhance oil crop production.

## Introduction

### Oilseeds have diverse compositions that provide valuable products for society

Food security affects 800 million people around the world (Singh 2024). Plant-based food, feed, and fuels will be increasingly important to sustain life as the world population reaches 8.5 billion by 2030 (The Sustainable Development Goals Report 2022). From an economic perspective, the value of seeds as a commodity is established by protein, oil, and carbohydrate composition. Here, even small changes in composition can result in large financial gains for the agriculture industry. As an example, for the 87 million acres in US annual soybean production (Vaiknoras 2023), an oil increase by 1% at the expense of an unvalued carbohydrate with 50 bushel(bu)/acre, assuming 60 pounds (lbs)/bu, 20% oil by composition, and a 3-year price per pound of oil ranging from $0.40 to $0.90 (Market Insider 2024), a farmer would produce a crop with $12–27/acre more value on Midwestern US fields, which would translate to a calculated $1 to $2 billion added revenue for the agriculture industry.

The primary constituent of vegetable oil is triacylglycerol (TAG) (Dyer et al. 2008; Baud and Lepiniec 2010; Allen et al. 2015; Sagun et al. 2023) with low levels of phospho- (Meng et al. 2014) and galactolipids (Sahaka et al. 2020). TAG contains acyl chains comprised of carbon–carbon bonds that store the energy needed for nutrition or for use as renewable fuels, plastics, lubricants, paints, and coatings that are currently derived from petroleum feed stocks (Metzger and Bornscheuer 2006; Cahoon et al. 2007; Durrett et al. 2008; Wayne et al. 2019; Napier and Betancor 2023). Oilseed TAGs predominantly contain 5 fatty acids, including palmitic (16:0) stearic (18:0), oleic (18:1), linoleic (18:2), and alpha-linolenic (18:3) acid, as summarized in **Table 1** (Dyer et al. 2008; Sharafi et al. 2015; Jain 2020).

**Table 1.** Global production of major oil seed crops in 2023/2024 along with oil content on a dry weight percent basis (% DW) and fatty acid composition as a percentage of the total (%).^a^

Oil seed	Production (10^6^ metric tons)	Oil (% DW)	Fatty acid composition (% of total)									
			Typical					Other				
			C16:0	C18:0	C18:1	C18:2	C18:3	C12:0	C14:0	C20:0	C22:0	C24:0
Soybean	398.21	20	10.6	4	23.3	53.7	7.6		0.1	0.3	0.3	
Rapeseed	87.44	40–45	3.6	1.5	61.6	21.7	9.6		0.1	0.6	0.3	0.2
Sunflower	55.08	35–42	7	4.5	18.7	67.5	0.8		0.1	0.4	0.7	0.2–0.3
Peanut	50.46	47–50	11.1	2.4	46.7	32	0				2.9	1.5
Cottonseed	41.46	25–35	21.6	2.6	18.6	54.4	0.7		0.3	0.3		
Palm kernel	20.71	50	8.5	2.4	15.4	2.5		47.8	16.3	0.2		
Copra	6.05	65–70	8.9	2.7	6.4	1.6		47.8	18.1	Tr		

^a^Data presented here are gathered from (Ulmasov et al. 2012; Dijkstra 2016; List 2016; Premnath et al. 2016; Kerr and Dunford 2018; Petrie et al. 2020; Aznar-Moreno et al. 2022; Li et al. 2022b).

Considerable diversity exists in biomass composition across different tissues and species. Lipid content ranges from less than 1% in peas and lentils to over 70% in walnuts and pecans and even up to 88% in palm mesocarp, while it remains below 5% in leaves (Lin and Oliver 2008; Allen et al. 2015). The diversity in TAG concentration suggests a significant opportunity to tailor plants with biotechnology or breeding efforts to meet increasing oil needs. In this update, we explore how vegetatively supplied nutrients and their conversion through biochemical pathway flux impacts seed storage reserve accumulation. We highlight the successful use of isotopic labeling combined with metabolic flux analysis (MFA) in uncovering nontraditional pathways for carbon and nitrogen utilization in oil seeds, as well as alternative carbon assimilation sites in plants. Additionally, we briefly discuss the recent engineering advances and potential future directions, including role of transcription factors (TFs), the manipulation of phytohormones, and technologies that together offer promising opportunities for engineering and breeding initiatives to improve oil content and composition.

### Seed metabolism and oil accumulation

#### Storage reserve production is limited by available sugars and amino acids over development

**Figure 1.**
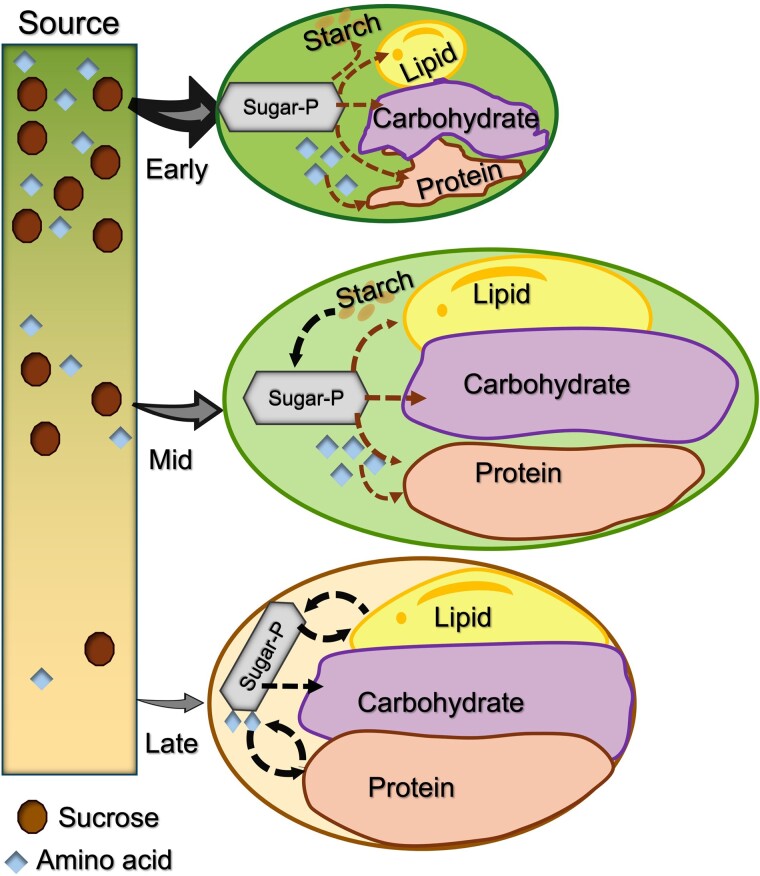
Storage reserve production depends on resource availability (example: soybean). Early seed development has access to a high level of maternal resources, including sucrose and amino acids. The incoming sucrose is metabolized to sugar phosphate (Sugar-P), or hexose phosphates more precisely, to make lipid, protein, starch, and other carbohydrates. Amino acids contribute to protein. Mid-seed development includes seed filling using vegetatively supplied resources, and carbon from starch turnover to produce Sugar-P for lipid, protein, and carbohydrates including oligosaccharides and cell wall polysaccharides. Few maternal resources are available at late seed development. Some existing reserves like lipid and protein are partially turned over to contribute carbon for carbohydrate biosynthesis. “Source” indicates photosynthesizing leaves and vegetative tissues. The green to yellow color gradient indicates the transition from young to senescence stages in the plant life cycle.

Maternal substrates available to developing seeds are finite (Pipolo et al. 2004; Hernández-Sebastià et al. 2005; Kambhampati et al. 2021). Consequently, the final composition of the seed is constrained by the resources it receives throughout development and the metabolic flux through pathways that transform these resources to seed reserves (Allen and Young 2013). As oilseeds contain a significant amount of stored protein, in addition to carbon they require ample nitrogen supplied from vegetative parts of the plant as amino acids (Rainbird et al. 1984; Fabre and Planchon 2000; Pipolo et al. 2004; Hernández-Sebastià et al. 2005; Allen et al. 2009; Truong et al. 2013; Koley et al. 2022). Photosynthetic carbon movement from source to sink tissues occurs predominantly via phloem (Thorne 1985) with sucrose (Weber et al. 1997a; Pegler et al. 2023) and a combination of several amino acids (Tegeder 2014; Besnard et al. 2018; Tegeder and Hammes 2018). Sugar transporters and their transport process have received considerable attention (Pegler et al. 2023), including sucrose transporters (SUT/SUC) (Weber et al. 1997b; Aldape et al. 2003; Baud et al. 2005), hexose symporters (STPs) (Weber et al. 1997b; Büttner 2010; Poschet et al. 2010; Pommerrenig et al. 2013; Rottmann et al. 2018), sucrose facilitators (SUFs) (Zhou et al. 2007), and “sugars will eventually be exported transporters” (SWEETs) (Chen et al. 2015; Yang et al. 2018; Fei et al. 2021). The movement and utilization of carbon as sugars and amino acids varies over the course of development and can dramatically impact final seed composition (Kambhampati et al. 2021). This remains an intriguing area to enhance yield or alter protein and oil. When the supply of carbon and nitrogen to soybeans was altered, protein content was dramatically impacted, changing from 14% to 47% of seed biomass, and was considered through modeled fluxes (Allen and Young 2013). Measurements of biomass in controlled field studies (Rotundo et al. 2011; Locke and Ramirez 2021) and other flux studies in seeds (Truong et al. 2013) have confirmed the change in seed protein as a consequence of maternal supply. The highest levels of sugars and amino acids available to developing seeds are present at early seed-filling stages and decreases during later stages (Kambhampati et al. 2021). Thus, efforts aimed at altering final seed composition, and potentially breaking negative associations between valued reserves like oil and protein, reported in soybean (Wilcox and Shibles 2001), rapeseed (Grami et al. 1977), sunflower (Li et al. 2017), and flax (Tavarini et al. 2016) may benefit from knowledge of the available concentrations over development (Kambhampati et al. 2021; Aznar-Moreno et al. 2022) (Fig. 1) as addressed below.

#### Late seed metabolism reduces commercial value and is a target for engineering

Maturation comprises 10% to 50% of the total time a seed develops (reviewed in Leprince et al. 2017). The temporal shifts in biomass composition of multiple oilseeds including Arabidopsis, rapeseed, and soybean indicate that lipid reserves are partially degraded during the maturation processes (Chia et al. 2005; Baud et al. 2008; Baud and Lepiniec 2009; Kambhampati et al. 2021). Lipid breakdown is likely prompted by the restricted nutrient supply in the later stages of development (Kambhampati et al. 2021) and is necessary to sustain ongoing metabolic demands, as indicated by levels of transcripts, enzymes, and metabolites (Baud and Graham 2006; Fait et al. 2006; Angelovici et al. 2010; Jones et al. 2010; Collakova et al. 2013; Galili et al. 2014; Kambhampati et al. 2020, 2021). As development proceeds, some oilseeds such as soybeans produce significant levels of raffinose family oligosaccharides (RFOs) (Hagely et al. 2013) and cell wall polysaccharides (Litterer et al. 2006). The RFOs are predicted to contribute to desiccation tolerance (Black et al. 1996; Obendorf 1997; Bailly et al. 2001), although results from soybean lines lacking raffinose synthase show no obvious phenotypic defects during maturation or germination (Dierking and Bilyeu 2008, 2009). Reducing the production of carbohydrates, such as RFOs, that have less commercial value in oilseeds could enhance oil content without negatively impacting protein levels, and this may be achievable by preventing lipid or other storage reserve breakdown during seed development (Aznar-Moreno et al. 2022). Related efforts have focused on a TAG lipase, Sugar Dependent Protein 1 (SDP1) (Kelly et al. 2013; Kim et al. 2014; Kanai et al. 2019; Azeez et al. 2022; Aznar-Moreno et al. 2022), though effort with other lipases including glycine-aspartate-serine-leucine (GDSL)-type esterases (Huang et al. 2015; Ding et al. 2023), Plastid Lipase1 (PLIP1) (Wang et al. 2017), and Monoacylglycerol lipase (MAGL) (Zhan et al. 2023) have been reported. Roles of other putative oil body-associated lipases (Eastmond 2004) in carbon partitioning between oil and carbohydrates remain to be explored. Seed-specific reductions in SDP1 through RNAi do not adversely affect seed maturation or germination (Kelly et al. 2013; Kim et al. 2014; Azeez et al. 2022; Aznar-Moreno et al. 2022) and can maintain protein levels similar to wild type (Aznar-Moreno et al. 2022), suggesting that the negative association between protein and oil may be more pliable than is currently thought.

**Figure 2.**
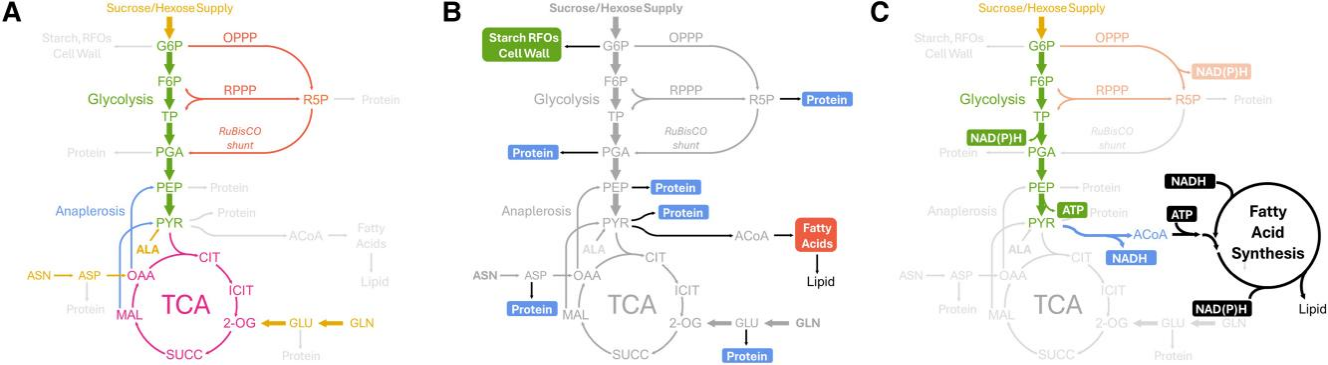
Key central carbon metabolic pathways that make seed storage reserves. **A)** Maternal resources like sucrose (the major carbohydrate source) and hexose and amino acids like alanine (ALA), asparagine (ASN), and glutamine (GLN) enter central metabolic pathways. Glycolysis, OPPP, reductive pentose phosphate pathway (RPPP), TCA, and anaplerosis reactions participate in producing oil, protein, and carbohydrates including starch, raffinose family oligosaccharides (RFOs), and cell wall polysaccharides (Cell Wall). Incoming maternal resources are colored yellow, glycolysis is green, PPP is red, TCA is purple, and anaplerosis is blue. Abbreviations: 2-OG, 2-oxoglutarate; ASP, aspartate; CIT, citrate; F6P, fructose-6-phosphate; G6P, glucose-6-phosphate; GLU, glutamate; ICIT, isocitrate; MAL, malate; OAA, oxaloacetate; PEP, phosphoenolpyruvate; PGA, phosphoglycerate; PYR, pyruvate; SUCC, succinate; TP, triose phosphate. **B)** At least six nodes in central metabolism contribute to protein precursors. Fatty acids for lipid biosynthesis, and carbohydrates are derived from a single precursor node in the network. **C)** Glycolysis is sufficient to produce ATP and reducing equivalents required for FAS. Conversion of glucose/hexose to two molecules of ACoA produces 2 NAD(P)H and 1 ATP per acetyl group, stoichiometrically equivalent to the requirement of fatty acid production. ACoA, acetyl-CoA.

To the extent that protein, carbohydrate, and lipid are concomitantly produced through metabolism, all require carbon derived from sugars and amino acids (Fig. 2A); however, the immediate precursors differ for each reserve. The distribution of precursors for protein (i.e. 20 amino acids) minimally requires six nodes in metabolism, whereas fatty acids are singly derived from acetyl-CoA building blocks and nucleotide sugar phosphates, used to synthesize carbohydrate polymers, are sourced from hexose phosphate in the chloroplast or cytosol (Allen et al. 2009; Lonien and Schwender 2009) (Fig. 2B). Though protein production can be limited by nitrogen, pyruvate is the substrate for five amino acids and acetyl-CoA for fatty acids (Fig. 2B). Understanding the regulation at the pyruvate node and methods to direct carbon to pyruvate may pose a strategy to enhance combined oil and protein levels, if other amino acid families are not significantly compromised (Morley et al. 2023).

### What have we learned from flux analysis in oilseed plants?

#### Plant seed-filling metabolism is distinct from animals and microbes

To understand how vegetatively supplied assimilates are used in developing seeds, flux analysis has focused on quantifying metabolic pathway use in seed filling during early and mid-development when oil and protein are synthesized. The consistent production of reserves through the early and mid-stages of development (Egli 2017) indicates steady-state metabolism and thus unchanging fluxes during the seed-filling period. MFA has quantitatively described atypical or unconventional metabolic pathway flows. These are distinct from textbook descriptions of central metabolism that are not specific to seed metabolism. For example, the production of fatty acids requires many acetyl-CoA building blocks that are derived from a combination of glycolytic flux and the pentose phosphate pathway with or without an oxidative component (i.e. OPPP). Though OPPP is thought necessary to produce the significant reductant needed for fatty acid synthesis (FAS), a number of green oilseeds utilize glycolysis and light without a complete requirement for NADPH production from the OPPP (Allen and Young 2013; Schwender et al. 2015). This minimizes oxidative steps that release carbon dioxide (CO_2_) to conserve available carbon. Importantly, the conversion of hexose phosphate to two acetyl-CoAs results in the concomitant production of 2 NAD(P)H and 1 ATP per acetyl group. This is stoichiometrically equivalent to the requirement for FAS. Thus, there is no absolute requirement for OPPP in the production of fatty acids in any living system, plant oilseed or otherwise (Fig. 2C), presuming glycolysis is present.

**Figure 3.**
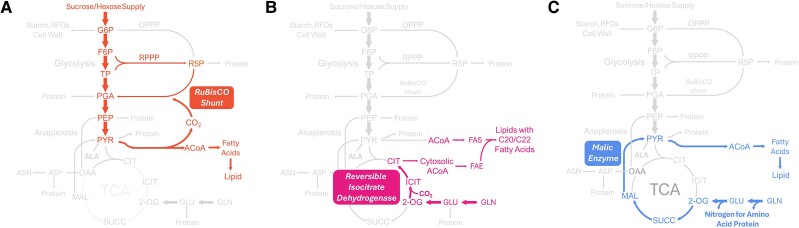
Nonconventional pathways in central metabolism provide carbon for fatty acids. **A)** Rubisco shunt fixes CO_2_ released during FAS in some green oilseeds. **B)** Reversible function of isocitrate dehydrogenase directs glutamine carbon toward the generation of citrate. Citrate is then metabolized to produce oxaloacetate and ACoA for fatty acid elongation (C20/C22). **C)** Glutamine contributes nitrogen for protein amino acid biosynthesis, resulting in co-production of organic acids that are partially processed by malic enzymes to pyruvate and ACoA for FAS.

Further, the use of Rubisco to reassimilate CO_2_ released during oil production in green oilseeds is well documented, initially in *Brassica napus* (Schwender et al. 2004; Junker et al. 2007; Schwender et al. 2015) and more recently in pennycress (Tsogtbaatar et al. 2020) and *Physaria* (Cocuron and Alonso 2024) and to a more limited extent in Camelina (Carey et al. 2020; Koley et al. 2022) and soybean (Allen et al. 2009) (Fig. 3A). Processes that conserve carbon, limiting the release of CO_2_ or that recover CO_2_, have improved conversion of carbon received by the seed into biomass, which is coined carbon conversion efficiency (CCE). Reported values for CCE have been recently reviewed (Sagun et al. 2023); importantly, the CCE is a consequence of storage reserve composition. Because the production of lipid requires precursors that are made using CO_2_-releasing steps such as pyruvate dehydrogenase, seeds that have high oil content will consequentially have a lower CCE if mechanisms are not in place to recover the respired CO_2_. With changes in the accumulation of biomass components during seed development, estimates of the CCE will vary temporally and may limit its usefulness if not considered specific to seed filling regimes (Kambhampati et al. 2021).

#### Seeds rely less on TCA-derived carbon skeletons for amino acid biosynthesis

Unlike *Escherichia coli* or other systems that receive inorganic nitrogen, seeds receive organic nitrogen primarily in the form of the amino acids glutamine, asparagine, and alanine. This has important consequences on the flux through the tricarboxylic acid (TCA) cycle and the resulting products. When ^13^C-glutamine is provided to oilseeds as a metabolic tracer, the labeling in resulting products was unanticipated and emphasized the differences in pathway use that are unconventional (summarized in Allen 2016a). For plants that produce very long chain fatty acids, reversible functioning isocitrate dehydrogenase can assimilate a limited amount of CO_2_ and subvert the TCA-based decarboxylation events to produce citrate. Citrate is exported from the mitochondria and through ATP-citrate lyase results in a source of acetyl-CoA for fatty acid elongation in rapeseed (Schwender et al. 2006), *Physaria* (Cocuron and Alonso 2024), and pennycress (Tsogtbaatar et al. 2020) (Fig. 3B).

In soybeans (Allen et al. 2009; Allen and Young 2013), the high level of protein produced capitalizes on the available glutamine nitrogen donated to other carbon skeletons to make the balance of 20 amino acids needed for protein production (Rainbird et al. 1984; Allen et al. 2015). Through this process, glutamine is twice deamidated, resulting in 2-oxoglutarate that is converted into other metabolites. Flux maps guided by ^13^C-labeled glutamine in soybean embryos revealed that 2-oxoglutarate is used to make malate. Then malic enzyme and pyruvate dehydrogenase are used in tandem to generate reductant and carbon that support FAS (Allen et al. 2009; Allen and Young 2013) (Fig. 3C). The value of malic enzyme for oil production has also been suggested in maize lines that naturally produce more lipid (Cocuron et al. 2019). Through a recent engineering approach, MFA results from soybean were validated (Morley et al. 2023; Schwender 2023). The study elucidated the contribution of subcellular malic enzyme activity to biomass composition. Transgenic alleles of malic enzyme were targeted to either the chloroplast or the extraplastidial compartments, resulting in changes in oil and free amino acid concentrations. In both cases, there was an increase in pyruvate-derived amino acids and a depletion of aspartate family amino acids. Malate was more heavily used by malic enzyme to make pyruvate relative to malate dehydrogenase–based production of oxaloacetate, the precursor for aspartate family amino acids. Increased pyruvate levels in transgenics suggested that further increase in oil will require enhancing steps that convert pyruvate to lipids and may indicate a rate-limiting role of acetyl-CoA carboxylase, the first committed step to lipid production (Salie and Thelen 2016; Ye et al. 2020). The subcellular location of malic enzyme activity also affected the degree of fatty acid polyunsaturation. This suggests that the co-production of reductant needed for desaturation events was impacted and may imply that malate valves (Selinski and Scheibe 2019; Dao et al. 2022; Morley et al. 2023) that rebalance reductant between subcellular locations were partially disrupted.

### Genetic engineering efforts to enhance oil content

#### Pushing carbon is a key to lipid production in seeds and leaves

The enhanced expression of malic enzyme results in more oil in seeds and is consistent with a push of carbon into pyruvate and fatty acid metabolism. As a strategy, pushing carbon has also been considered in engineered leaves (Vanhercke et al. 2017) and stems (Parajuli et al. 2020) to increase lipid levels. Many TFs are integral to this “push” strategy (Yang et al. 2022a; Sagun et al. 2023), including the master regulator WRINKLED 1 (WRI1) (Cernac and Benning 2004; Baud and Lepiniec 2010; Bates et al. 2013; Ma et al. 2013; van Erp et al. 2014; Kong et al. 2020). WRI1 has been extensively studied through endogenous and heterologous expression of homologs resulting in enhanced seed oil quantity and quality in *Lepidium campestre* (Ivarson et al. 2017), *Glycine max* (Chen et al. 2018), *Linum usitiassimum* (Li et al. 2022a), and *Arabidopsis thaliana* (Lim et al. 2022). Additionally, other transcriptional regulators have shown promise in enhancing seed oil yield either independently or by regulating WRI1 and present opportunities for further analysis. Some of the most tantalizing candidates are discussed in the review by Sagun et al. (2023).

Interestingly, when high-oil tobacco leaves (Vanhercke et al. 2017) were evaluated with isotopically nonstationary MFA (INST-MFA), they showed increased flux catalyzed by malic enzyme (Chu et al. 2022) as a consequence of engineering other steps. However, the same was not true in fluxes through Arabidopsis seeds (Lonien and Schwender 2009). This indicates that the role of TFs is both tissue and species specific. For the most part, direct downstream target genes of many of these TFs that impact oil synthesis remain to be fully identified. Given its role as a “master regulator” that controls the expression of many genes important for the conversion of sugars into fatty acids, more attention has been paid to WRI1. For example, in vitro DNA binding studies in soybean (Chen et al. 2018) and genome-wide studies with a phylogenetic footprinting approach in Brassicaceae family (Kuczynski et al. 2022) and soybean WRI1 binding sites (Jo et al. 2024) indicated interaction with the AW-box and CNC-box cis acting element. Such interactions regulate the expression of genes involved in fatty acid production, elongation, desaturation, and exit from plastid along with predicted targets in glycolysis and the PPP. While many studies of the impact of TFs could be explored with MFA, to date reports are limited to those involving WRI1 (Lonien and Schwender 2009; Chu et al. 2022). Other concepts, originating from efforts to coexpress diacylglycerol acyltransferase DGAT (Behera et al. 2023), oleosin protein (Fan et al. 2013; Winichayakul et al. 2013), or other lipid-packaging systems (James et al. 2010) and regulation by SEIPINS (Cai et al. 2015) as part of “pull” or “packaging” approaches, have also strongly impacted oil production and are promising strategies netting significant gains in seeds and leaves (van Erp et al. 2014; Vanhercke et al. 2014, 2019) but are beyond the scope of this review.

#### Maternal sucrose supply governs oil seed carbon push

The push of carbon into oilseeds originates from supplies of sucrose and photosynthetic carbon assimilation in leaves has received attention in recent years. Leaves of oilseeds including Arabidopsis (Ma et al. 2014), tobacco (Chu et al. 2022; Fu et al. 2023), and camelina (Xu et al. 2021) have been mapped with INST-MFA to analyze photosynthesis that can limit crop productivity (Koley et al. 2024). These studies collectively identified changes in photorespiration that occurred with high light acclimation (Ma et al. 2014), the export of amino acids from photorespiration (Fu et al. 2023), and the unexpected oxidative metabolism of carbon (i.e. OPP metabolism) that occurs concomitantly with carbon assimilation in photosynthesis (Xu et al. 2021, 2022). However, there are no MFA models directly tying flux from leaves to developing seeds across multiple organs and tissues, and until recently (Koley et al. 2022) no multiorgan flux maps existed for any living system.

#### Silique tissues are photosynthetic and contribute synergistically to seed biomass

Though leaves are a primary source of carbon assimilation, studies in many plants indicate that pod walls and siliques can make a significant contribution to seed biomass (Atkins et al. 1977; Imaizumi et al. 1997; Furbank et al. 2004; Pengelly et al. 2011; AuBuchon-Elder et al. 2020; Koley et al. 2022). Oilseeds such as camelina grow with siliques high above the canopy, resulting in unencumbered access to light. In addition, siliques are produced temporally, immediately before the seeds. At the time of seed-set, many leaves have withered and fallen away. These observations suggested that silique photosynthesis may contribute to the developing cotyledons in camelina (Koley et al. 2022). MFA showed that a significant percentage of carbon in the seeds (33% to 45%) was derived from silique-based photosynthetic assimilation of CO_2_ with the remainder coming from leaves. The siliques provide a “just-in-time” delivery of photoassimilates to the seeds contained within the pod. Unlike leaves, siliques do not transfer carbon to other plant parts (Koley et al. 2022) and may enhance the viable seed number. Further, the proximity of the silique may imply reduced pressure differentials between the source and sink, potentially lowering the transport cost relative to a long-distance translocation of sugars. This study represents a first multiorgan metabolic flux map for any biological system, plant or otherwise. Relatedly, a recent study with field grown soybean indicated that pod and seed photosynthesis contribute about 9% of daily canopy carbon gain with a 13% to 14% contribution to seed weight (Cho et al. 2023), building on the “green” seed potential of soybean assessed by MFA (Allen et al. 2009) and emphasizing that our understanding of what contributes to seed yield is incomplete and would benefit from additional MFA studies that include more organs and tissues.

### Advances in technology and methods will unlock new possibilities

#### Stable isotopes with modern technologies and software can complement traditional radiolabeling evaluations of flux

Though considerable progress has been made to quantify central metabolism by computational MFA in oilseeds, much of what we know about FAS and lipid assembly, exchange, and breakdown have relied on radiolabeling studies to deduce flux information (e.g. Pollard et al. 2015; reviewed in Allen et al. 2015). Seminal findings documented the acyl exchange that explains high polyunsaturation in TAG (Bates et al. 2009) and have suggested that the rapid labeling in phosphatidylcholine may indicate the involvement of this lipid in shuttling acyl chains to the endoplasmic reticulum (Tjellström et al. 2012; Allen 2016a; Karki et al. 2019). These studies are complementary to stable isotope investigations. Recent efforts with stable isotopes (Kambhampati et al. 2024) indicate the added value that isotopologue quantification using mass spectrometry (MS) can contribute to addressing important questions in lipid biology. MS measurements to quantify lipids, that is, lipidomics (Welti et al. 2007; Romsdahl et al. 2022) and imaging-MS (Romsdahl et al. 2021; Horn and Chapman 2024) or nuclear magnetic resonance (Borisjuk et al. 2023), define differences in lipids or other components that provide an indication of the metabolic phenotype. In some cases, this includes spatial resolution that could complement multiorgan investigations or identify phenotypic variation at the cellular level within seeds. Isotopes can assess the actively produced lipids in different tissues with this purpose in mind (Romsdahl et al. 2021). The use of stable isotopes and modern technologies such as the application of high-resolution MS (HRMS) (Allen 2016a; Allen and Young 2020) remain a largely untapped strategy to quantitatively describe lipid metabolism and will continue to advance the field with improvements in instrument resolution and new data analysis platforms (Kambhampati et al. 2024). Similarly, absolute quantification techniques, including aqua-multiple reaction monitoring (Ahsan et al. 2018) and methods to measure intermediates in FAS including acyl-acyl carrier proteins, (i.e. acyl-ACPs) (Nam et al. 2020; Jenkins et al. 2021) and their labeling (Chu et al. 2022), provide examples of technological advances that will contribute to quantitatively explain lipid production and breakdown dynamics.

#### Acyl-ACP quantification indicates fatty acid metabolism is incompletely described

ACPs act as intermediates in FAS. Like other intermediates, the levels of ACPs are not strictly tied to flux through FAS; however, several reports to date suggest that tissues producing more oil generally have a higher level of measured ACPs (Chu et al. 2022) and greater enzymatic activities (Ohlrogge and Kuo 1984). When the tissue is not producing a significant amount of lipid, then the levels of the individual acyl-ACPs can be lower and more challenging to quantify (Xu et al. 2023). During development acyl-ACPs decline when oil and protein are being made (Morley et al. 2023). Measurements of acyl-ACPs as a readout to understand oil production have been considered in several oilseeds (Bates et al. 2014; Kim et al. 2015) and algae (Msanne et al. 2021). The unanticipated presence of polyunsaturated acyl-ACPs indicates our understanding of FAS is incomplete (Nam et al. 2020). Polyunsaturated acyl-ACPs could reflect the breakdown of chloroplast lipids in seeds, as they transition from green photosynthetically capable organs to non-green storage compartments taking advantage of acyl-ACP synthetases (Koo et al. 2005). The breakdown of lipids, through recycling with development (Kambhampati et al. 2021) that may include beta oxidation of fatty acids (Koley, Allen, unpublished data) or for lipid remodeling as recently depicted (Parchuri et al. 2024), remains a largely unexplored area in metabolism that could be deduced from the combination of isotopic labeling and quantitative techniques such as MFA.

#### Mechanistic evaluation of hormones holds potential for oil yield and improving fatty acid content

One of the other areas that could benefit from the application of quantitative approaches is the exploration of phytohormones. Studies underlying the ability of phytohormones to alter fatty acid composition and oil biosynthesis are either restricted to exogenous application of these hormones like indole-3-acetic acid, cytokinin 6-benzylaminopurine (Talukdar et al. 2022), abscisic acid (Jadhav et al. 2008), or altered gene expression of hormone precursors (Kant et al. 2015). Limited reports are available indicating the regulation of fatty acid and seed oil content by phytohormones via signaling cascades (Thien Nguyen et al. 2016) impacting TFs associated with the “push” pathway and GDSL-type Seed Fatty Acid Reducer gene (Chen et al. 2012). However, in most cases these mechanisms remain incompletely quantified if not uncharacterized.

In combination, the coordinated regulation of FAS by developmental regulators such as phytohormones is an open area with chemical factors affecting metabolite and transcripts during seed development. Since hormones like abscisic acid play a critical role in stress responses, integrating developmental and environmental factors with changes in metabolic flux will potentially result in discoveries that enhance plant resilience in future climates and could additionally contribute to higher oil–yielding species.

## Conclusion

The network of central metabolism that supports production of protein, oil, and carbohydrates is flexible and operates with different throughputs for seeds than other tissues or systems (Allen 2016b), in part because of the maternal provisions that seeds receive from vegetative tissue, but also because of the evolved role as a storage organ. Our understanding of how seed metabolism operates continues to be shaped by recent studies and assumptions originating from the animal or microbial kingdoms may not be applicable. To advance the production of seeds with improved compositions, future studies must consider temporal aspects during seed development when the composition is changing, the balance of carbon allocated to different reserves and their subcomponents, and the synergies between tissues of the plant and how they are impacted by changes in the environment. Such consideration can explain what metabolic or developmental functions the turnover of lipids over seed growth and maturation fulfills and if they are required for viability (Outstanding Questions). The production and degradation of lipids that may appear futile could in fact be a necessary rapid sensitive response to changes in the environment that has been conserved throughout time. New technologies that more precisely and quantitatively evaluate aspects of metabolism are emerging. These are crucial to provide types of comprehensive descriptions that are common for gene expression and protein. Though most modern-day labeling techniques involve labeled carbon (^14^C or ^13^C), an abundance of stable isotope choices including labeled hydrogen (^2^H), oxygen (^17^O,^18^O), nitrogen (^15^N), and sulfur (^33^S, ^36^S) are now commercially available and can be rigorously quantified with MS-based technologies including HRMS and can be adapted for INST-MFA (Allen 2016a; Allen and Young 2020). This would help to elucidate fluxes that otherwise might be challenging to estimate (Kambhampati et al. 2024) and enable the rational development of pathways for carbon redistribution in agriculturally beneficial ways to enhance value-added traits in oil seed crops (Azeez et al. 2022; Aznar-Moreno et al. 2022; Morley et al. 2023; Sagun et al. 2023).

**Advances**

- Descriptions of seed metabolism focus on accumulation of storage reserves overlooking reserve breakdown, including the degradation of oil, that reduces final seed value and presents an opportunity for engineering.
- The TCA in plant seeds operates differently than in other tissues or living systems, resulting in distinct roles for isocitrate dehydrogenase and malic enzyme in oil production based on MFA. Malic enzyme contribution was recently validated through a genetic engineering approach.
- Flux analyses in leaves and photosynthetically active reproductive structures (i.e. siliques and pods) using INST-MFA emphasize that the sources of carbon for developing seeds are incompletely understood.
- Stable isotopic labeling from multiple elements coupled with HRMS and new software tools provide an opportunity for more comprehensive MFA.

**Outstanding questions**

- Does carbon resulting from lipid breakdown late in seed development contribute to biosynthesis of other seed storage reserves, prepare the seed for dormancy and future germination, or what roles does it serve?
- Can the negative association between protein and oil be decoupled through engineering efforts focused on temporal differences in seed metabolism and limiting carbohydrate production?
- What contribution can reproductive structures make to the seed carbon economy? How much of the carbon that is released as CO_2_ to make precursors for fatty acid biosynthesis in embryos and cotyledons is recovered within the reproductive organs under different environments and in different species?
- How can TFs and phytohormone targets be better leveraged across development to positively influence seed composition?

## Data Availability

No data was generated as a part of this review article.

## Dive Curated Terms

The following phenotypic, genotypic, and functional terms are of significance to the work described in this paper:
